# Observed size distribution changes in American lobsters over a 12-year period in southwestern Nova Scotia, Canada

**DOI:** 10.1371/journal.pone.0295402

**Published:** 2023-12-07

**Authors:** Svenja Koepper, Crawford W. Revie, Henrik Stryhn, Shannon Scott-Tibbetts, Krishna K. Thakur

**Affiliations:** 1 Department of Health Management, Atlantic Veterinary College, University of Prince Edward Island, Charlottetown, Canada; 2 Department of Computer and Information Sciences, University of Strathclyde, Glasgow, United Kingdom; 3 Fishermen and Scientists Research Society, Halifax, Canada; Havforskningsinstituttet, NORWAY

## Abstract

Size distribution and size frequency information of American lobsters (*Homarus americanus*) are often used to help estimate the age distributions, and reproductive output for the species and to guide the determination of appropriate minimum legal sizes for the fishery. This study used truncated linear regression models to estimate the effects of sampling year, sampling month, lobster sex and water depth on the lobster size. A dataset of almost 130,000 trap–caught lobsters from the two most important lobster fishing areas of Atlantic Canada collected over a 12-year period (2004–2015) was analyzed. It was shown that truncated models can help to account for biases due to the trap sampling method from vessels and from wharf samplings. There were significant annual and seasonal changes in size distribution, and data collected outside the fishing season showed a significant increase in carapace length in 2014 and 2015, potentially reflecting a northward shift of the range of lobster populations due to more favourable settlement and recruitment habitats. Size also increased in late summer, likely due to moult. Our results demonstrated that landed lobsters, especially females, were smaller than the predicted size-at-maturity in the region (96.5 mm carapace length), which could have long-term repercussions for the stock’s reproductive potential.

## Introduction

For the commercially fished American lobster, their size distribution is one of the most important life-history traits to determine the reproductive potential and growth of the population [1, 2]. Monitoring these distributions is therefore important to develop adequate 2021fishing regulations, given that 50–100% of the legal-sized population is fished in southwestern Nova Scotia [3, 4]. Size distributions in crustaceans can be influenced by fisheries, temperature, substrate, currents, recruitment, or adult migration [5–7]. Previous studies have recorded spatial and temporal size variability in Atlantic Canada, but the definite causes are still not well understood [7, 8].

The size of lobsters directly determines whether animals can be fished because the Canadian lobster fishery is subject to minimum legal size (MLS) regulations and traps must have escape vents to let undersized animals exit traps. This measure increases the number of eggs and the weight yield per recruit and it also ensures that the market is supplied with a range of differently-sized lobsters [9]. Lobster fishing areas (LFAs) vary in their MLS with the Bras D’Or region (Nova Scotia) having the largest MLS (84 mm CL) and the southern Gulf of St. Lawrence and Northumberland Straight (Prince Edward Island) having the smallest (73 mm CL) [9–11]. Fisheries management decisions on the MLS are informed by the size-at-maturity (SaM) which differs regionally and represents the size at which 50% of females in the population reach maturity [9]. Setting the MLS above the SaM aims to ensure that at least half the female population has a chance to spawn before capture [12, 13]. However, in some LFAs, including LFA 33 and 34, the MLS is set below SaM meaning that most of the females will not reproduce before being recruited into the fishery. Declines in female SaM have been observed in the northwestern Atlantic but it is unclear whether these changes are due to rising temperatures or evolutionary adaptation to fishing pressure on larger animals [14, 15]. For male lobsters, SaM can only be assessed by invasive methods or by the relative size of the crusher claw which is not often collected during standard surveys [16]. The growth rate in lobsters is closely linked to the surrounding water temperatures, with higher temperatures expediting the metabolism and stimulating moulting [16]. While moult increments vary by location, it has been shown that carapace lengths increase per moult (in mm) are greater in warmer than in colder regions [16]. Therefore, increasing water temperatures in recent years could also affect lobster size distributions and the SaM [5, 12, 14, 15]. However, evidence for this relationship remains difficult to obtain and in Atlantic Canada declines in SaM were not consistent with rising temperatures [14].

Size is a widely used variable for age determination in crustaceans due to the lack of other well-established methods, such as otolith analysis in teleost fish [2, 17]. Unlike vertebrates and bivalves, crustaceans shed their exoskeleton with every moult so that no hard structures with growth bands remain to indicate the animal’s age [18]. For highly commercial species, such as lobster, age distributions are a baseline for modelling population dynamics which requires life-history data such as size, longevity, and growth rate [2].

Environmental variables, together with different capture methods, can influence observed size distributions, making consistent sampling essential to obtain meaningful size data. Lobster data from commercial fishing vessels can be more easily collected than extensive trawl surveys, which usually take place only for a few weeks during certain times of the year. Collecting samples from fisheries would enable scientists to access data almost year-round. Extending knowledge on how lobster population dynamics are shaped during the fishing season may elucidate how exploitation impacts local lobster stocks. However, the use of traps fitted with escape vents and the manual discard of small individuals or berried females, and minimum legal sizes will introduce biases to the dataset in commercial fisheries. This study aims to account for size biases by using truncated regression models to estimate the seasonal and annual changes in size distributions of trap-caught lobsters as well as determine any differences in size distributions by water depth and between lobster sex in the most productive lobster fishing regions of Atlantic Canada in southwestern Nova Scotia (LFA 33 and 34).

## Materials and methods

### Data collection

For a detailed description of sampling procedures and the dataset, see the published data descriptor in Koepper et al. [19]. This study uses data from lobsters that were sampled in LFA 33 and 34 only, due to the highest data coverage in these regions. Briefly, data were collected in the framework of the Atlantic Lobster Moult and Quality project implemented through the Atlantic Veterinary College (University of Prince Edward Island) and the Fishermen and Scientists Research Society from 2004–2015 to monitor shell quality and lobster moult cycles. While lobsters were collected in a standardized manner, traps with escape vents were used, and sampling methods differed based on off-season sampling (from vessels) or within-season sampling (from the wharf). Therefore, due to the fishing regulations and methods, lobster size is a truncated variable and affected by the time of sampling. During the fishing season (DFS), lobsters were sampled from the wharf and only legal-sized lobsters were sampled (commercial traps with escape vents), truncating the size data at the minimum legal size (82.5 mm CL). Outside of the fishing season (OFS), lobsters were sampled at sea directly from traps (commercial traps with escape vents) and all sizes were included in the sampling. Berried females were not landed on the wharf for DFS sampling and were not targeted either for OFS vessel sampling according to the study protocol [19]. Therefore, berried females were excluded from this analysis. In both LFA 33 and 34, the fishing season starts at the end of November (the last Monday of the month) and lasts until May 31^st^. In LFA 33, most of the fishery takes place within 15 km from shore, whereas in LFA 34 offshore regions are also fished [20].

### Statistical analysis

To account for different truncation cut-offs the dataset was divided into two parts which were analyzed separately: with OFS samples being from June to November and DFS samples from December to May. Both subsets were analyzed using truncated linear regression models (-truncreg-) in Stata (v. 17, Stata Corp, 2022) with the cubic-root transformed lobster size (mm CL) as the outcome to meet model assumptions as indicated by a Box-Cox analysis (OFS: theta = 0.4, DFS: theta = 0.3). The used models considered lobsters as independent data points. To account for lobsters in the same sampling event to be more similar in size, robust standard errors clustered on sampling event were used which assume between-cluster independence. The truncation cut-offs for size were chosen at 70.0 mm CL for OFS and at 82.5 mm CL for DFS, according to the size at which a lobster can escape through escape vents and the minimum legal size respectively [10, 21]. Observations below these size thresholds were truncated by the model and not used for estimation, as the -truncreg- command estimates the underlying nontruncated distribution only based on data above the truncation cutoff. Forward model building was used in both approaches, with predictors and interactions below the 0.05 significance level being kept in the final model. For OFS data, the final model included the predictors sampling month, moult stage and two two-way interactions between the LFA and sampling year, as well as lobster sex and water depth. The final model for DFS data included one two-way interaction between lobster sex and water depth, as well as the predictors sampling year, sampling month, LFA and moult stage. The final regression models for OFS and DFS are listed below (*β*_0_ = model intercept, *β*_1–5_ = regression coefficients (including interactions and their sets of parameters), *ε* = error):

$$OFS:\;{\sqrt[{3}]{Size}}_{OFS}=\beta_{0}+\beta_{1}(LFA*Year)+\beta_{2}(Sex*Water\;depth)+\beta_{3}(Month)+\beta_{4}(Moult\;stage)+\varepsilon$$ (1)

$$DFS:\;{\sqrt[{3}]{Size}}_{DFS}=\beta_{0}+\beta_{1}(LFA)+\beta_{2}(Year)+\beta_{3}(Sex*Water\;depth)+\beta_{4}(Month)+\beta_{5}(Moult\;stage)+\varepsilon$$ (2)

For both models, residuals were calculated by subtracting the fitted values from the observed values for the cubic-root transformed lobster size. Assumptions for normality and homoscedasticity were assessed by plotting the model residuals. To plot the respective effects of sampling time, LFA, water depth and lobster sex, all other predictive factors included in the model were fixed to meaningful values. When comparing size distributions across the sampling months and between sex and water depth, the sampling year was fixed to 2014 as it was the most recent and LFA was fixed to 34, as data was collected over more sampling locations there and overall has higher lobster landings than LFA 33 [10, 19]. Model outputs for other scenarios (e.g. where variables were fixed to other values) are presented in S1 and S2 Figs in S1 File. Sea surface temperature was significant only in the OFS model but not in the DFS model. Sea surface temperature is not as relevant to benthic species as bottom temperature and provided similar model input as sampling month in our analysis. To include the same fixed effects and keep models comparable it was decided to exclude sea surface temperature from the model-building process. To compare annual and seasonal differences, post-hoc pairwise comparisons were conducted between sampling months and years and adjusted for multiple comparisons (Bonferroni correction) (S3 and S4 Tables in S1 File). Model outputs were back-transformed to the original scale for visualization.

## Results

### Descriptive statistics

Descriptive statistics for all variables included in the dataset were published in Koepper et al. [19]. For convenience, sampling frequencies and descriptive statistics for categorical and continuous variables relevant for this study are summarized in Tables 1 and 2. Overall, there were 906 sampling events (OFS = 699, DFS = 207) with an average of 119 (SD = 62.83) lobsters sampled per event. From a total of 128,015 sampled lobsters, 103,868 observations were included in the final models (OFS = 75,775; 508 were truncated; DFS = 28,093; 1,772 were truncated) due to missing values for the variable water depth.

**Table 1:** Overview and sampling frequencies of the categorical variables lobster sex and moult stage of lobsters sampled from 2004–2015.

Variable	Season	N	NA	Sampling frequencies			
				**Male**		**Female**	
**Sex**	All	128,015	20	68,778		59,217	
	OFS	88,506	17	46,538		41,951	
	DFS	39,509	3	22,240		17,266	
				**Postmoult/Intermolt**	**Passive premoult**		**Active Premoult**
**Moult stage**	All	128,015	174	71,348	47,009		9,484
	OFS	88,506	161	49,882	31,567		6,896
	DFS	39,509	13	21,466	15,442		2,588

N = number of lobsters sampled per factor variable, NA missing values. Active premoult = moult stages D1, D2; passive premoult = moult stages D0 (after Aiken [22]).

**Table 2:** Overview and descriptive statistics of the continuous outcome variable size as well as water depth and sea surface temperature.

Variable	Season	N	NA	Mean	1%	Median	99%
**Size**	All	128,015	15	89.2	71	88	130
	OFS	88,506	13	87.7	70	86	129
	DFS	39,509	2	92.7	80	90	132
**Water depth**	All	128,015	21,669	22.1	4.0	12.2	122.6
	OFS	88,506	12,039	22.6	4.0	18.3	122.6
	DFS	39,509	9,630	20.9	4.0	12.2	122.6
**Sea surface temperature**	All	128,015	14,778	9.6	0.9	11.9	15.9
	OFS	88,506	9,205	11.9	6.9	11.9	16.4
	DFS	39,509	5,573	4.3	0.5	4.5	9.8

N = number of lobsters sampled per factor variable. NA = missing values. *for truncated regression analysis, lobster size was truncated at 70 mm CL (OFS) and 82.5 mm CL (DFS).

Both OFS and DFS model outputs and parameters can be found in supplementary S1 and S2 Tables in S1 File.

### Annual and seasonal patterns in lobster size

**Fig 1:**
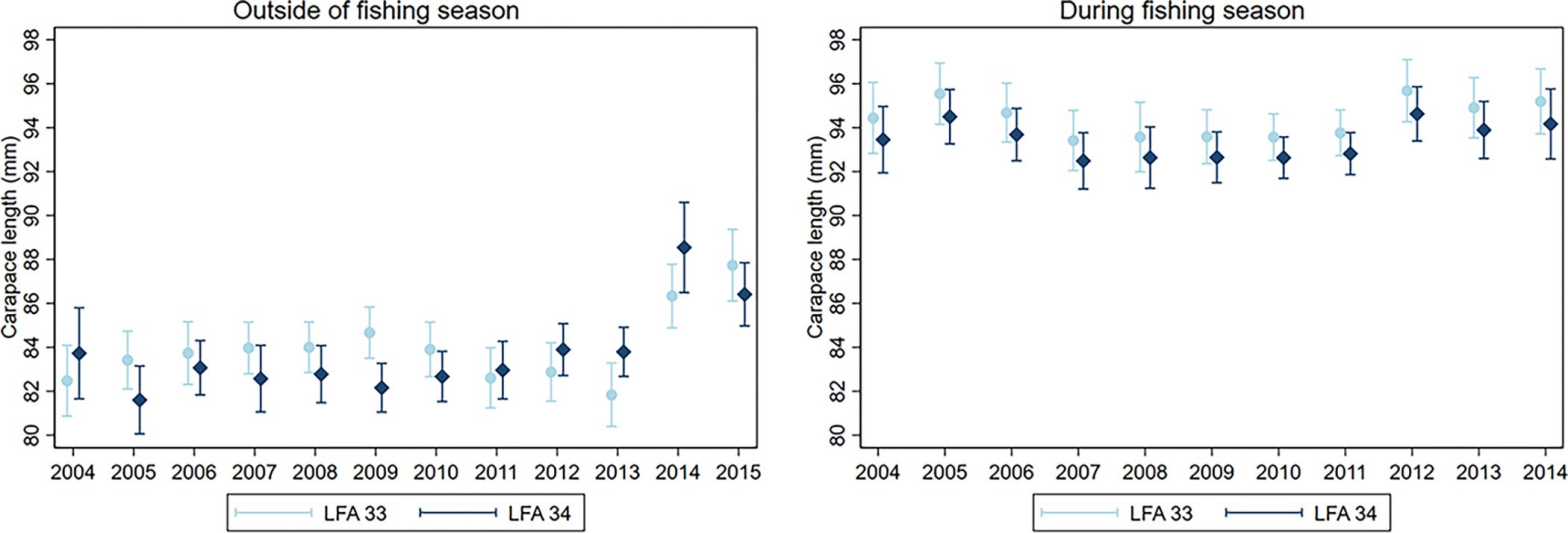
Estimated lobster sizes (with 95% confidence intervals) over the sampling period from 2004 to 2015. Size was dependent on the LFAs outside the fishing season (OFS, Jun-Nov, left panel) and during the fishing season (DFS, Dec-May, right panel) according to truncated regression models. Model predictors not displayed on the graphs were fixed to: month = June (OFS)/ May (DFS), sex = male, moult stage = intermoult, water depth = 10 m. Note that no data was available during the fishing season in 2015.

When visualizing the effects of year and LFA, sampling months were fixed to May (DFS model) and June (OFS model) to consider adjacent months in the two models. Lobster sex was fixed to either females or males and water depth was fixed to either shallower (10 m) or deeper (50 m) waters, allowing the assessment of size differences by sex and sampling depths. The moult stage was set to only include intermoult lobsters as they represented most sampled lobsters and are the most relevant to fisheries. Based on a significant interaction term between LFA and sampling year, the estimated lobster size between years depended on the LFA outside the fishing season (Fig 1). From 2004 to 2013, mean lobster size varied between 81.6–84.6 mm CL in both LFAs but in 2014 and 2015, the estimated lobster sizes increased to 86.3–88.5 mm CL. Post-hoc pairwise comparisons (Bonferroni corrected) confirmed that lobsters in the last two years (2014–2015) of the survey were significantly larger than in the first nine years (2004 to 2013) (S3 Table in S1 File). In 2009, sampled lobsters were larger in LFA 33 as compared to LFA 34 (p = 0.006, pairwise comparison). During the fishing season, our model estimated significant interannual fluctuation between the sampling years (p < 0.0001, Wald test) but the interaction between LFA and sampling year was not significant. Over the study period, lobster size fluctuated annually and did not reflect the pattern observed in OFS data. Lobsters sampled in 2010 were significantly smaller in 2010 compared to 2005 (p = 0.017), 2012 (p = 0.021), and 2014 (p = 0.006) in pairwise comparisons. During the fishing season, no data was available for 2015. Overall, lobsters were larger during the fishing season than outside the fishing season, with sizes ranging from 92.5 to 95.2 mm CL, compared to 81.6 to 88.5 mm CL outside the fishing season.

**Fig 2:**
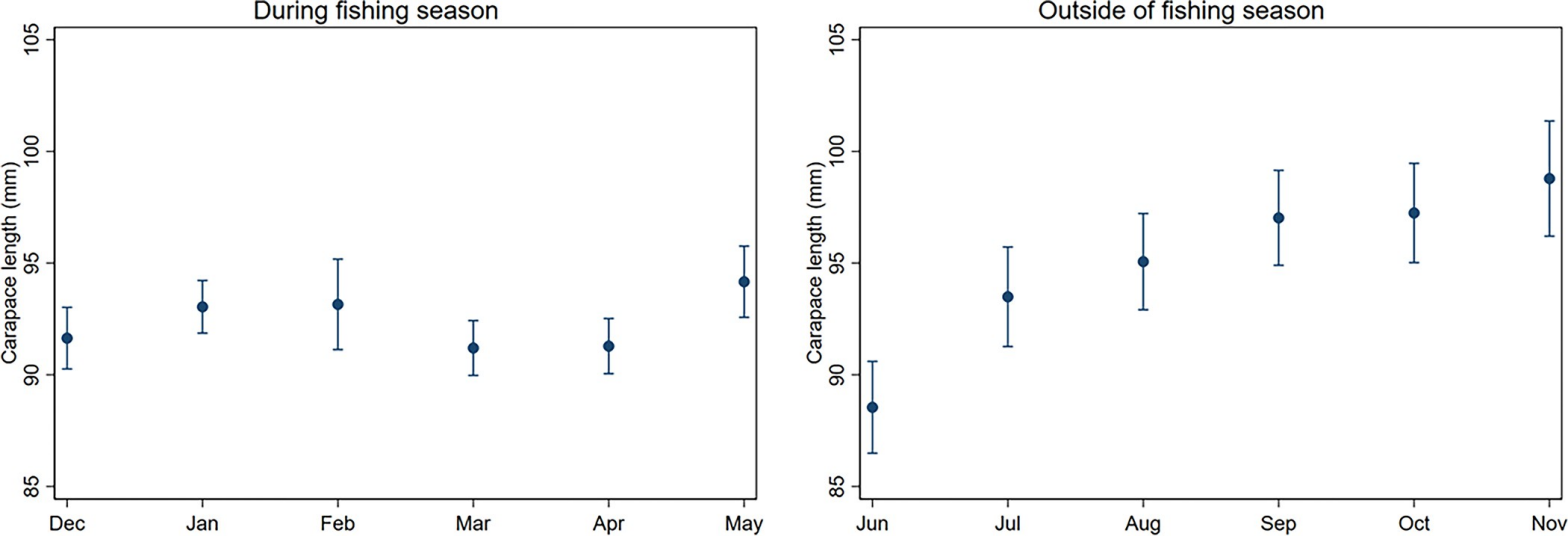
Estimated lobster sizes (with 95% confidence intervals) over the sampling year. Size displayed for either outside the fishing season (OFS, Jun-Nov, left panel) and during the fishing season (DFS, Dec-May, right panel) using truncated regression models. Model predictors not displayed on the graphs were fixed to: year = 2014, LFA = 34, sex = male, moult stage = intermoult, water depth = 10 m.

When comparing size distributions across the sampling months and between sex and water depth, the sampling year was fixed to 2014 as it was the most recent and LFA was fixed to 34, as data was collected over more sampling locations there and overall has higher lobster landings than LFA 33 [10, 19]. Model outputs for other scenarios (e.g. where variables were fixed to other values) are presented in S1 and S2 Figs in S1 File. Regarding seasonal changes in lobster size distributions, outside the fishing season, our model estimated a size increase from June (88.5 mm CL) to November (98.8 mm CL) with lobsters in June being smaller compared to all subsequent months (p < 0.0001, Bonferroni corrected pairwise comparisons) (Fig 2). During fishing season, lobster size differed significantly between sampling months and ranged from 91.2 mm (March) to 94.2 mm CL (May). Lobsters were significantly larger in May than in March (p < 0.0001), April (p < 0.0001) and December (p < 0.0001).

### Effects of water depth and lobster sex

**Fig 3:**
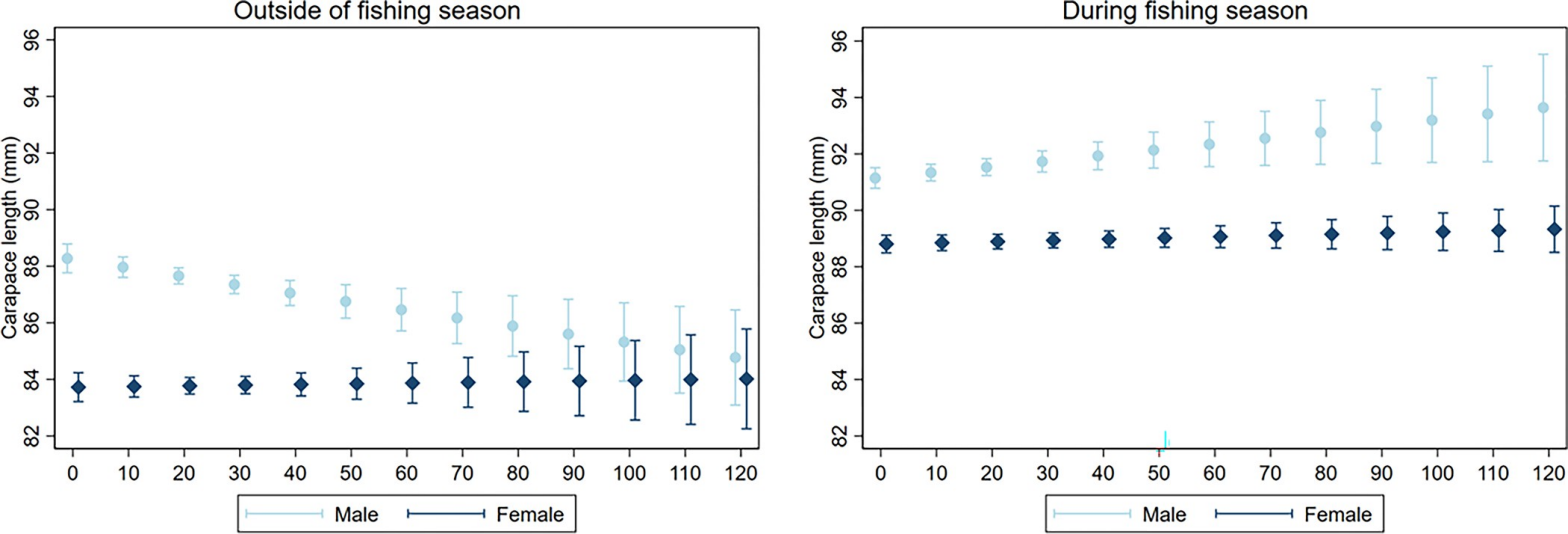
Estimated lobster size (with 95% confidence intervals) for male and female lobsters at different sampling depths (m). Size estimates displayed for either during the fishing season (OFS, December–May, left panel) and during the fishing season (DFS, June–November, right panel) using truncated regression models. Model predictors not displayed on the graphs were fixed to: year = 2014, month = June (OFS)/ May (DFS), LFA = 34, moult stage = intermoult.

Overall, more male lobsters (54%) were sampled than females (46%) and in OFS samples this difference between males was smaller (males: 53%, females: 47%) than in DFS samples (males: 56%, females: 44%). In both OFS and DFS models, the effect of sampling depth on lobster size estimates depended on lobster sex. Outside the fishing season, the female size estimates did not change from shallower to deeper waters, but male size estimates decreased from 88.0 (10 m) to 84.8 mm CL (120 m) in June (Fig 3, left panel) indicating that it is more likely to sample larger males in shallow waters. Below 80 m water depth, there was no estimated size difference between male and female lobsters. During the fishing season from December to May, the interaction between water depth and lobster sex was borderline significant (p = 0.046) which was also indicated by almost parallel lines in the interaction plot (Fig 3, right panel). Here, the model estimated larger males in deeper waters in May (91.3 mm CL at 10 m vs. 93.6 mm CL at 120 m) but only a slight size increase was observed for female lobsters (88.8 mm CL at 10 m vs. 91.2 mm CL at 120 m). Regarding the moult stages, lobsters in active and passive premoult were smaller than lobsters in the intermoult stage both during and outside the fishing season.

## Discussion

We present a comprehensive analysis of observed lobster size distributions obtained using commercial traps from LFAs 33 and 34, the most important lobster fishing areas in Atlantic Canada. A longitudinal dataset such as the one used in this study can provide meaningful size estimates and we evaluated how trap and fishery induced size truncation during the fishing season (DFS) and outside the fishing season (OFS) can be compensated by using truncated linear regression models.

When comparing OFS and DFS lobster size estimates across the sampling years, lobsters during the fishing season were larger than lobsters caught outside the fishing season when looking at the adjacent months May (94.2 mm CL, DFS) and June (88.5 mm CL, OFS). Because we used truncated regression models, we assume the size biases due to the OFS and DFS sampling methods (minimum legal size vs. undersized lobsters escaping from traps) to have been accounted for, meaning that these estimates should reflect the true size means of the sampled population. Still, after the end of the fishing season in June, we showed that the carapace length of sampled lobsters was almost 5 mm smaller than those caught in May. These results are comparable to trawl and trap surveys in LFA 34 right after the fishing season in July and during the fishing season in November–May conducted by DFO (Department for Fisheries and Oceans Canada) during 2008 and 2010 [23]. The mean lobster size in midshore areas ranged from 90–96 mm CL in November (at-sea sampling from commercial vessels) and from 80–82 mm CL in July (trawl survey) [23]. Several factors could drive this observed drop in lobster size: Firstly, there may still be unaccounted fisheries factors present, namely, fishers targeting mainly larger individuals and setting traps at locations where they are more likely to trap larger animals. Outside the fishing season, the sampling on chartered vessels or the trawls in the DFO survey may not have used exactly the same locations for the traps. However, the DFO trawl data showed a sharp decrease in the abundance of lobsters above the MLS limit in July [23]. This suggests that larger animals were likely fished during the fishing season and with fewer large lobsters present in the area, smaller individuals may have higher foraging activity and are therefore more likely to encounter traps, hence decreasing the mean size frequency [24, 25]. This also could account for the size difference between the two seasons in the annual comparisons. Smaller lobsters sampled in June could also be due to new recruits moulting into the target fishery as soon as environmental conditions become favourable in the spring [16]. Younger lobsters tend to moult more frequently than older, larger lobsters, and a spring moult of these smaller animals could also be reflected in our data. The size estimates from this study for the July 2008–2010 time period (82–84 mm CL) were comparable to the DFO trawl survey in the same LFA at that time (80–82 mm CL) albeit slightly larger [23]. This suggests that the truncated models were able to estimate the true mean of the population outside the fishing season. Unfortunately, during the fishing season (November–May) no trawl data for comparison was available, but the estimated size data collected from wharves (in this study) was comparable to data collected at sea [23].

Annually, OFS lobster size depended on LFAs but only in 2009, were lobsters larger in LFA 33 than in LFA 34. While these two LFAs are adjacent, the environmental conditions such as ocean currents and seafloor habitat are likely different which could influence population dynamics including size [7, 26]. Higher fishing pressure in LFA 34 compared to LFA 33 may also be a factor in the observed difference in size distributions between regions. LFA 34 had higher landings and fishing pressure had increased during the study period as compared to LFA 33 where it had decreased [3, 4]. Higher fishing pressure would usually be associated with a higher removal of males, which in turn could affect the size distributions in heavily fished lobster populations. However, similar size estimates in both LFAs may indicate that oceanographic effects or fisheries did not influence size distributions in this study greatly. We saw an increase in size frequency over the course of the study period. Pinsky and Fogarty [27] have shown that the American lobster range is shifting northward from southern New England towards the Gulf of Maine and the adjacent fishing regions in Atlantic Canada. This has been associated with warming temperatures leading to unfavourable environmental conditions in the southern range of the species affecting larval dispersal, post-larval settlement, recruitment and survival [28]. While these population mechanisms played a role in the size increase in 2014 and 2015, these effects would be difficult to observe over just a few years. A northward movement of larger, mature lobsters has not been documented yet, but Campbell and Stasko [29] have shown extended movements of adult American lobsters from the Bay of Fundy with some lobsters migrating over 100 km within a one- or two-year timespan. More tag-and-release studies are necessary to test the possibility of individual northward movement in the Gulf of Maine and Nova Scotia. Higher settlement and better recruitment of lobsters in the studies areas would be beneficial for the Canadian lobster fishery, and result in higher catches in the future if these trends persisted. Regarding DFS samples, lobster size estimates showed interannual variation but no obvious increasing or decreasing patterns in size. Previously, size fluctuations both over the years and during the year have been reported from similar studies in Canada and are most likely due to yearly temperature fluctuations or annual changes in recruitment and settlement [7, 30].

An increase in lobster size over the summer and into fall was apparent from our model outputs. This coincides with the moult cycle of American lobster, where adults moult into larger size classes, therefore increasing the frequency of larger lobsters in the population [18]. This pattern has already been shown by Thakur et al. [31], who looked at shell quality as an indicator of lobster moult in the same dataset. It demonstrates that seasonal changes in lobster size distribution could also be used as moult indicators in longitudinal datasets. During the fishing season, the size estimates fluctuated unidirectionally between December and April, although size could have been expected to decrease due to the removal of larger animals due to fishing, previously observed in Canadian lobster populations by Tremblay and Smith [25]. However, especially in regions with high fishing pressure, such as LFA 33 and 34, size distributions can vary more than in times of no fishing (OFS data) or compared to unfished regions [7]. These variations may prevent us from seeing clear trends during the fishing season.

Water depth is an important variable for lobster distribution as it influences migration, sex ratios, spawning and recruitment [30, 32]. Lobster depth profiles should therefore to some extent be size-specific as shallow waters serve for the settlement of postlarval and juvenile animals whereas deeper waters are inhabited by larger adult lobsters that are less susceptible to predation [33]. Offshore and also inshore adult lobsters in the Gulf of Maine and the Bay of Fundy also migrate seasonally to deeper waters in the fall and back to shallow waters in spring [29, 33, 34]. Male lobsters prefer waters closer to the shore in the summer because it enhances their growth due to higher temperatures [35]. Consequently, our results showed that it was more likely to sample larger males in shallow waters in the OFS summer months. During the fishing season, there was a size difference between male and female lobsters, with males being consistently larger than females, even in deeper waters. During fishing months, i.e. winter months, larger males were captured in deeper waters as compared to smaller males in shallow waters. Previous studies have shown that it is more likely to catch larger lobsters at deeper depths, especially in the winter when larger adults take part in seasonal offshore migration [7, 30, 33]. Both outside and during the fishing season the size estimates for female lobsters did not change much over the depth gradient, although the DFS model showed a slight increase in female lobster size at deeper depths. The effect of water depths on female lobster size may not be as obvious here because the sampling protocol of this study did not target egg-bearing -hence larger- females who prefer deeper waters for egg development [36]. Accordingly, size trends for larger fecund female lobsters are not as easy to detect in our data set.

Models have shown that a lower SaM has negative effects on fecundity in unfished populations, while the opposite effect is predicted in fished populations [14]. If lobsters become mature at a smaller size, their chance of spawning multiple times before harvest increases [14]. The size at which 50% of the females in the population mature (CL_50_) has declined drastically over the past decades due to intense fishing efforts [14]. Accordingly, the CL_50_ was reported as 96.5 mm CL in LFA 34 in 2013 with the size of the smallest mature female (CL_min_) as 77.5 mm in LFA 33 and 72.5 mm in LFA 34 in 2013 [14]. The size estimates from our truncated model during the fishing season (representing landed lobsters) generally do not exceed 95 mm CL, with an overall mean of 92.6 mm CL which reflects that the MLS of 82.5 mm CL is smaller than the CL_50_. We acknowledge that our dataset did not consider berried females which ultimately skewed the observations towards immature females. In a similar study, [30] showed that in the same LFAs, most females were trapped below 91 mm CL and therefore below the estimated SaM (traps without escape vents). Removing females below the CL_50_, i.e. could have repercussions for the reproductive output in these regions as these females likely did not have a chance to spawn. Importantly, available size at maturity data for these regions have not been updated within the last decade. Hence, it is important that up-to-date information on female size at maturity be collected to confirm whether the CL_50_ decline in LFA 34 continues. This would be necessary to make informed decisions on potential MLS adjustments.

Considering the high exploitation rates, changing environmental conditions, and declines in CL_50_/CL_min_, the monitoring of lobster size patterns is important for inferring the reproductive potential of the stock and for making informed decisions on fisheries regulations. Conducting research on lobster size distributions and other life-history traits is substantially more feasible from commercial vessels or wharves, compared to more extensive trawl surveys. In this study, we showed that using truncated regression models can be a relatively easy approach to account for trap and fishery-induced size biases, estimating similar size patterns compared to trawl and at-sea sampling in the same areas and time spans. Implementing truncated regression models on data collected from commercial traps, e.g throughout the fishing season, will result in valuable information that can be used to make informed stock management decisions which ultimately benefit the sustainability of the Canadian lobster fisheries in the region. Based on the research question, we would recommend sampling from vessels as it also enables the recording of larger ovigerous females, a major limitation that this dataset presented.

## Supporting information

S1 File (DOCX)
