# Supplementary material for: Observed size distribution changes in American lobsters over a 12-year period in southwestern Nova Scotia, Canada
Source: PLoS One. 2023 Dec 7;18(12):e0295402. doi: 10.1371/journal.pone.0295402 (PMC10703322; doi:10.1371/journal.pone.0295402)
Supplement: S1 File — (DOCX) [file pone.0295402.s001.docx]

Supplementary materials

Observed size distribution changes in American lobsters over a 12-year period in southwestern Nova Scotia, Canada

Svenja Koepper^1*^, Crawford W. Revie^2^, Henrik Stryhn^1^, Shannon Scott-Tibbetts^3^, Krishna K. Thakur^1^

^1^Department of Health Management, Atlantic Veterinary College, University of Prince Edward Island, Charlottetown C1A 4P3 PE, Canada

^2^Department of Computer and Information Sciences, University of Strathclyde, Glasgow G1 1XQ, UK

^3^Fishermen and Scientist Research Society, Halifax, NS B3M 4H4, Canada

**S1 Table.** **Parameters and outputs from the truncated linear regression models for data collected outside the fishing season (OFS).** Includes variables and categories of variables (if applicable), their coefficients, robust standard errors (SE), and p-values. AIC = Akaike information criterion.

| **Outside fishing season** | | | | |
| --- | --- | --- | --- | --- |
| **N** | | 75,775 | | |
| **N (truncated)** | | 508 | | |
| **Sampling events** | | 699 | | |
| **Log Pseudolikelihood** | | -58,078.5 | | |
| **AIC** | | 116,227.0 | | |
| **Variables** | | **Coefficient** | **Robust SE** | **P-value** |
| Sex | Female | -0.2402 | 0.0115 | 0.000 |
| Water depth |  | -0.0015 | 0.0005 | 0.002 |
| Sex#Water depth | | 0.0016 | 0.0004 | 0.000 |
| LFA | 34 | 0.0746 | 0.0688 | 0.278 |
| Year | 2005 | 0.0566 | 0.0521 | 0.000 |
|  | 2006 | 0.0751 | 0.0535 |  |
|  | 2007 | 0.0885 | 0.0482 |  |
|  | 2008 | 0.0903 | 0.0460 |  |
|  | 2009 | 0.1280 | 0.0466 |  |
|  | 2010 | 0.0847 | 0.0509 |  |
|  | 2011 | 0.0081 | 0.0549 |  |
|  | 2012 | 0.0243 | 0.0527 |  |
|  | 2013 | -0.0401 | 0.0584 |  |
|  | 2014 | 0.2169 | 0.0499 |  |
|  | 2015 | 0.2872 | 0.0512 |  |
| LFA#Year | 34#2005 | -0.1866 | 0.0854 | 0.000 |
|  | 34#2006 | -0.1139 | 0.0786 |  |
|  | 34#2007 | -0.1573 | 0.0774 |  |
|  | 34#2008 | -0.1466 | 0.0757 |  |
|  | 34#2009 | -0.2225 | 0.0748 |  |
|  | 34#2010 | -0.1476 | 0.0771 |  |
|  | 34#2011 | -0.0533 | 0.0820 |  |
|  | 34#2012 | -0.0147 | 0.0778 |  |
|  | 34#2013 | 0.0439 | 0.0815 |  |
|  | 34#2014 | 0.0345 | 0.0827 |  |
|  | 34#2015 | -0.1410 | 0.0741 |  |
| Month | July | 0.2165 | 0.0339 | 0.000 |
|  | August | 0.2789 | 0.0329 |  |
|  | September | 0.3531 | 0.0296 |  |
|  | October | 0.3611 | 0.0306 |  |
|  | November | 0.4166 | 0.0380 |  |
| Moult stage | Passive premoult | -0.1010 | 0.0099 | 0.000 |
|  | Active premoult | -0.2545 | 0.0159 |  |
| Intercept | | 2.3405 | 0.0510 | 0.000 |

**S2 Table. Parameters and outputs from the truncated linear regression models for data collected during the fishing season (DFS).** Includes variables and categories of variables (if applicable), their coefficients, robust standard errors (SE), and p-values. AIC = Akaike information criterion.

| **During fishing season** | | | | |
| --- | --- | --- | --- | --- |
| **N** | | 28,093 | | |
| **N (truncated)** | | 1,772 | | |
| **Sampling events** | | 207 | | |
| **Log Pseudolikelihood** | | -25,813.72 | | |
| **AIC** | | 51,673.4 | | |
| **Variables** | | **Coefficient** | **Robust SE** | **P-value** |
| Sex | Female | -0.2038 | 0.0170 | 0.000 |
| Water depth | | 0.0015 | 0.0006 | 0.013 |
| Sex#Water depth | | -0.0011 | 0.0005 | 0.046 |
| LFA | 34 | -0.0644 | 0.0235 | 0.006 |
| Year | 2005 | 0.0680 | 0.0498 | 0.000 |
|  | 2006 | 0.0153 | 0.0500 |  |
|  | 2007 | -0.0670 | 0.0524 |  |
|  | 2008 | -0.0564 | 0.0594 |  |
|  | 2009 | -0.0555 | 0.0513 |  |
|  | 2010 | -0.0566 | 0.0489 |  |
|  | 2011 | -0.0438 | 0.0514 |  |
|  | 2012 | 0.0762 | 0.0420 |  |
|  | 2013 | 0.0292 | 0.0565 |  |
|  | 2014 | 0.0469 | 0.0608 |  |
| Month | Jan | 0.1013 | 0.0392 | 0.000 |
|  | Feb | 0.1088 | 0.0593 |  |
|  | Mar | -0.0339 | 0.0411 |  |
|  | Apr | -0.0274 | 0.0313 |  |
|  | May | 0.1761 | 0.0335 |  |
| Moult stage | Passive premoult | -0.0713 | 0.0164 | 0.000 |
|  | Active premoult | -0.2148 | 0.0271 |  |
| Intercept | | 2.1007 | 0.0398 | 0.000 |

**S3 Table. Pairwise comparisons (Bonferroni corrected) of estimated lobster sizes between sampling years 2014 and 2015 to the previous years (OFS) and between all sampling years (DFS) in LFA 33 and 34**. Non-significant pairwise comparisons are highlighted in bold. DFS = during fishing season, OFS outside fishing season.

| **Season** | **Comparison** | **Contrast** | **SE** | **z** | **P** | **95% CI** | |
| --- | --- | --- | --- | --- | --- | --- | --- |
| OFS | 33 2014) vs (33 2004) | 0.21689 | 0.04988 | 4.35 | 0.004 | 0.03014 | 0.40364 |
|  | (33 2015) vs (33 2004) | 0.28719 | 0.05125 | 5.60 | 0.000 | 0.09533 | 0.47905 |
|  | (34 2014) vs (33 2004) | 0.32608 | 0.05779 | 5.64 | 0.000 | 0.10972 | 0.54244 |
|  | (34 2015) vs (33 2004) | 0.22088 | 0.04884 | 4.52 | 0.002 | 0.03802 | 0.40374 |
|  | 33 2014) vs (33 2005) | 0.16028 | 0.03984 | 4.02 | 0.016 | 0.01111 | 0.30945 |
|  | (33 2015) vs (33 2005) | 0.23058 | 0.04252 | 5.42 | 0.000 | 0.07139 | 0.38977 |
|  | (34 2014) vs (33 2005) | 0.26947 | 0.05041 | 5.35 | 0.000 | 0.08073 | 0.45821 |
|  | (34 2015) vs (33 2005) | 0.16427 | 0.03953 | 4.16 | 0.009 | 0.01627 | 0.31227 |
|  | **(33 2014) vs (33 2006)** | **0.1418** | **0.04074** | **3.48** | **0.138** | **-0.0107** | **0.29435** |
|  | (33 2015) vs (33 2006) | 0.21211 | 0.04383 | 4.84 | 0.000 | 0.04803 | 0.37619 |
|  | 34 2014) vs (33 2006) | 0.251 | 0.05112 | 4.91 | 0.000 | 0.05962 | 0.44237 |
|  | **(34 2015) vs (33 2006)** | **0.14579** | **0.04094** | **3.56** | **0.102** | **-0.0075** | **0.29907** |
|  | **(33 2014) vs (33 2007)** | **0.12835** | **0.03556** | **3.61** | **0.085** | **-0.0048** | **0.26149** |
|  | (33 2015) vs (33 2007) | 0.19866 | 0.03841 | 5.17 | 0.000 | 0.05485 | 0.34246 |
|  | (33 2014) vs (33 2008) | 0.12656 | 0.03315 | 3.82 | 0.037 | 0.00243 | 0.25068 |
|  | (33 2015) vs (33 2008) | 0.19686 | 0.0356 | 5.53 | 0.000 | 0.06359 | 0.33013 |
|  | (34 2014) vs (33 2008) | 0.23575 | 0.04466 | 5.28 | 0.000 | 0.06856 | 0.40294 |
|  | (34 2015) vs (33 2008) | 0.13055 | 0.03182 | 4.10 | 0.011 | 0.01141 | 0.24969 |
|  | **(33 2014) vs (33 2009)** | **0.08884** | **0.03376** | **2.63** | **1.000** | **-0.0376** | **0.21524** |
|  | (33 2015) vs (33 2009) | 0.15915 | 0.03677 | 4.33 | 0.004 | 0.02149 | 0.29681 |
|  | (34 2014) vs (33 2009) | 0.19804 | 0.04545 | 4.36 | 0.004 | 0.02789 | 0.36818 |
|  | **(34 2015) vs (33 2009)** | **0.09284** | **0.03291** | **2.82** | **1.000** | **-0.0304** | **0.21604** |
|  | **(33 2014) vs (33 2010)** | **0.13217** | **0.03838** | **3.44** | **0.158** | **-0.0115** | **0.27586** |
|  | (33 2015) vs (33 2010) | 0.20248 | 0.04105 | 4.93 | 0.000 | 0.04877 | 0.35618 |
|  | (34 2014) vs (33 2010) | 0.24137 | 0.04911 | 4.92 | 0.000 | 0.05752 | 0.42521 |
|  | **(34 2015) vs (33 2010)** | **0.13617** | **0.03786** | **3.60** | **0.089** | **-0.0056** | **0.27792** |
|  | (33 2014) vs (33 2011) | 0.20879 | 0.04383 | 4.76 | 0.001 | 0.0447 | 0.37288 |
|  | (33 2015) vs (33 2011) | 0.27909 | 0.04614 | 6.05 | 0.000 | 0.10634 | 0.45185 |
|  | (34 2014) vs (33 2011) | 0.31798 | 0.05346 | 5.95 | 0.000 | 0.11784 | 0.51813 |
|  | (34 2015) vs (33 2011) | 0.21278 | 0.04339 | 4.90 | 0.000 | 0.05033 | 0.37523 |
|  | (33 2014) vs (33 2012) | 0.1926 | 0.04202 | 4.58 | 0.001 | 0.03529 | 0.34992 |
|  | (33 2015) vs (33 2012) | 0.26291 | 0.04359 | 6.03 | 0.000 | 0.09973 | 0.42609 |
|  | (34 2014) vs (33 2012) | 0.3018 | 0.05149 | 5.86 | 0.000 | 0.10901 | 0.49459 |
|  | (34 2015) vs (33 2012) | 0.1966 | 0.04086 | 4.81 | 0.000 | 0.0436 | 0.34959 |
|  | (33 2014) vs (33 2013) | 0.25702 | 0.04905 | 5.24 | 0.000 | 0.0734 | 0.44065 |
|  | (33 2015) vs (33 2013) | 0.32733 | 0.05105 | 6.41 | 0.000 | 0.13619 | 0.51846 |
|  | (34 2014) vs (33 2013) | 0.36622 | 0.05746 | 6.37 | 0.000 | 0.15109 | 0.58134 |
|  | (34 2015) vs (33 2013) | 0.26102 | 0.0484 | 5.39 | 0.000 | 0.07982 | 0.44221 |
|  | (33 2015) vs (33 2014) | 0.0703 | 0.03619 | 1.94 | 1.000 | -0.0652 | 0.20578 |
|  | (34 2004) vs (33 2014) | -0.1422 | 0.05662 | -2.51 | 1.000 | -0.3542 | 0.06974 |
|  | (34 2005) vs (33 2014) | -0.2722 | 0.05053 | -5.39 | 0.000 | -0.4614 | -0.083 |
|  | (34 2006) vs (33 2014) | -0.181 | 0.03504 | -5.17 | 0.000 | -0.3122 | -0.0498 |
|  | (34 2007) vs (33 2014) | -0.211 | 0.03817 | -5.53 | 0.000 | -0.3539 | -0.0681 |
|  | (34 2008) vs (33 2014) | -0.1985 | 0.03899 | -5.09 | 0.000 | -0.3445 | -0.0525 |
|  | (34 2009) vs (33 2014) | -0.2367 | 0.037 | -6.40 | 0.000 | -0.3752 | -0.0982 |
|  | (34 2010) vs (33 2014) | -0.2051 | 0.03571 | -5.74 | 0.000 | -0.3388 | -0.0714 |
|  | (34 2011) vs (33 2014) | -0.1875 | 0.04018 | -4.66 | 0.001 | -0.3379 | -0.037 |
|  | (34 2012) vs (33 2014) | -0.1326 | 0.03474 | -3.82 | 0.037 | -0.2627 | -0.0026 |
|  | (34 2013) vs (33 2014) | -0.1385 | 0.03354 | -4.13 | 0.010 | -0.2641 | -0.0129 |
|  | (34 2014) vs (33 2014) | 0.10919 | 0.04606 | 2.37 | 1.000 | -0.0632 | 0.28162 |
|  | (34 2015) vs (33 2014) | 0.00399 | 0.03053 | 0.13 | 1.000 | -0.1103 | 0.1183 |
|  | (34 2004) vs (33 2015) | -0.2125 | 0.05825 | -3.65 | 0.073 | -0.4306 | 0.00554 |
|  | (34 2005) vs (33 2015) | -0.3425 | 0.0516 | -6.64 | 0.000 | -0.5357 | -0.1493 |
|  | (34 2006) vs (33 2015) | -0.2513 | 0.03732 | -6.73 | 0.000 | -0.391 | -0.1116 |
|  | (34 2007) vs (33 2015) | -0.2813 | 0.03999 | -7.04 | 0.000 | -0.431 | -0.1316 |
|  | (34 2008) vs (33 2015) | -0.2688 | 0.04114 | -6.53 | 0.000 | -0.4228 | -0.1148 |
|  | (34 2009) vs (33 2015) | -0.307 | 0.03964 | -7.74 | 0.000 | -0.4554 | -0.1586 |
|  | (34 2010) vs (33 2015) | -0.2754 | 0.03874 | -7.11 | 0.000 | -0.4204 | -0.1304 |
|  | (34 2011) vs (33 2015) | -0.2578 | 0.04269 | -6.04 | 0.000 | -0.4176 | -0.0979 |
|  | (34 2012) vs (33 2015) | -0.2029 | 0.03691 | -5.50 | 0.000 | -0.3411 | -0.0648 |
|  | (34 2013) vs (33 2015) | -0.2088 | 0.03627 | -5.76 | 0.000 | -0.3446 | -0.073 |
|  | (34 2014) vs (33 2015) | 0.03889 | 0.04825 | 0.81 | 1.000 | -0.1417 | 0.21952 |
|  | (34 2015) vs (33 2015) | -0.0663 | 0.0324 | -2.05 | 1.000 | -0.1876 | 0.05498 |
|  | (34 2005) vs (34 2004) | -0.13 | 0.06653 | -1.95 | 1.000 | -0.379 | 0.11912 |
|  | (34 2014) vs (34 2004) | 0.25143 | 0.06353 | 3.96 | 0.021 | 0.01359 | 0.48928 |
|  | (34 2015) vs (34 2004) | 0.14623 | 0.05341 | 2.74 | 1.000 | -0.0537 | 0.3462 |
|  | (34 2014) vs (34 2005) | 0.38139 | 0.05825 | 6.55 | 0.000 | 0.16332 | 0.59946 |
|  | (34 2015) vs (34 2005) | 0.27619 | 0.04707 | 5.87 | 0.000 | 0.09997 | 0.4524 |
|  | (34 2014) vs (34 2006) | 0.2902 | 0.04644 | 6.25 | 0.000 | 0.11633 | 0.46407 |
|  | (34 2015) vs (34 2006) | 0.185 | 0.03138 | 5.89 | 0.000 | 0.0675 | 0.3025 |
|  | (34 2014) vs (34 2007) | 0.32021 | 0.0482 | 6.64 | 0.000 | 0.13977 | 0.50065 |
|  | (34 2015) vs (34 2007) | 0.21501 | 0.03414 | 6.30 | 0.000 | 0.0872 | 0.34282 |
|  | (34 2014) vs (34 2008) | 0.30769 | 0.04984 | 6.17 | 0.000 | 0.1211 | 0.49429 |
|  | (34 2015) vs (34 2008) | 0.20249 | 0.03624 | 5.59 | 0.000 | 0.06682 | 0.33816 |
|  | (34 2014) vs (34 2009) | 0.34591 | 0.04802 | 7.20 | 0.000 | 0.16612 | 0.5257 |
|  | (34 2015) vs (34 2009) | 0.24071 | 0.03392 | 7.10 | 0.000 | 0.11373 | 0.36769 |
|  | (34 2014) vs (34 2010) | 0.31431 | 0.04744 | 6.63 | 0.000 | 0.1367 | 0.49191 |
|  | (34 2015) vs (34 2010) | 0.20911 | 0.03351 | 6.24 | 0.000 | 0.08365 | 0.33456 |
|  | (34 2014) vs (34 2011) | 0.29665 | 0.05056 | 5.87 | 0.000 | 0.10734 | 0.48595 |
|  | (34 2015) vs (34 2011) | 0.19144 | 0.03774 | 5.07 | 0.000 | 0.05014 | 0.33274 |
|  | (34 2014) vs (34 2012) | 0.24183 | 0.04602 | 5.26 | 0.000 | 0.06954 | 0.41412 |
|  | (34 2015) vs (34 2012) | 0.13663 | 0.03139 | 4.35 | 0.004 | 0.01911 | 0.25414 |
|  | (34 2014) vs (34 2013) | 0.24769 | 0.04546 | 5.45 | 0.000 | 0.07749 | 0.41788 |
|  | (34 2015) vs (34 2013) | 0.14248 | 0.03061 | 4.65 | 0.001 | 0.02788 | 0.25709 |
|  | **(34 2015) vs (34 2014)** | **-0.1052** | **0.04412** | **-2.38** | **1.000** | **-0.2704** | **0.05998** |
| DFS | (34 2012) vs (34 2010) | 0.14681 | 0.03755 | 3.91 | 0.021 | 0.00793 | 0.28569 |
|  | (34 2010) vs (34 2005) | -0.1537 | 0.03873 | -3.97 | 0.017 | -0.297 | -0.0105 |
|  | (34 2010) vs (33 2014) | -0.1831 | 0.04363 | -4.2 | 0.006 | -0.3445 | -0.0217 |

Table S4: Pairwise comparisons (Bonferroni corrected) of estimated lobster sizes between sampling months outside fishing season (OFS, Jun - Nov) and during fishing season (DFS, Dec - May).

| **Season** | **Comparison** | **Contrast** | **SE** | **z** | **P** | **95% CI** | |
| --- | --- | --- | --- | --- | --- | --- | --- |
| OFS | Jul vs Jun | 0.216 | 0.03 | 6.38 | 0.000 | 0.117 | 0.316 |
|  | Aug vs Jun | 0.279 | 0.03 | 8.49 | 0.000 | 0.182 | 0.375 |
|  | Sep vs Jun | 0.353 | 0.03 | 11.94 | 0.000 | 0.266 | 0.440 |
|  | Oct vs Jun | 0.361 | 0.03 | 11.79 | 0.000 | 0.271 | 0.451 |
|  | Nov vs Jun | 0.417 | 0.04 | 10.95 | 0.000 | 0.305 | 0.528 |
|  | Aug vs Jul | 0.062 | 0.03 | 2.33 | 0.295 | -0.016 | 0.141 |
|  | Sep vs Jul | 0.137 | 0.02 | 5.7 | 0.000 | 0.066 | 0.207 |
|  | Oct vs Jul | 0.145 | 0.03 | 5.72 | 0.000 | 0.070 | 0.219 |
|  | Nov vs Jul | 0.200 | 0.03 | 6.23 | 0.000 | 0.106 | 0.294 |
|  | Sep vs Aug | 0.074 | 0.02 | 3.62 | 0.004 | 0.014 | 0.134 |
|  | Oct vs Aug | 0.082 | 0.02 | 3.69 | 0.003 | 0.017 | 0.148 |
|  | Nov vs Aug | 0.138 | 0.03 | 4.72 | 0.000 | 0.052 | 0.223 |
|  | Oct vs Sep | 0.008 | 0.02 | 0.44 | 1.000 | -0.046 | 0.062 |
|  | Nov vs Sep | 0.064 | 0.03 | 2.42 | 0.235 | -0.014 | 0.141 |
|  | Nov vs Oct | 0.055 | 0.03 | 2.02 | 0.648 | -0.025 | 0.136 |
| DFS | Jan vs Dec | 0.097 | 0.04 | 2.34 | 0.292 | -0.025 | 0.219 |
|  | Feb vs Dec | 0.111 | 0.06 | 1.76 | 1.000 | -0.073 | 0.295 |
|  | Mar vs Dec | -0.034 | 0.04 | -0.85 | 1.000 | -0.152 | 0.083 |
|  | Apr vs Dec | -0.029 | 0.03 | -0.91 | 1.000 | -0.124 | 0.065 |
|  | May vs Dec | 0.174 | 0.04 | 4.96 | 0.000 | 0.071 | 0.277 |
|  | Feb vs Jan | 0.014 | 0.07 | 0.2 | 1.000 | -0.184 | 0.212 |
|  | Mar vs Jan | -0.131 | 0.04 | -2.92 | 0.052 | -0.263 | 0.001 |
|  | Apr vs Jan | -0.126 | 0.04 | -3.3 | 0.015 | -0.239 | -0.014 |
|  | May vs Jan | 0.077 | 0.04 | 2.01 | 0.673 | -0.036 | 0.190 |
|  | Mar vs Feb | -0.145 | 0.07 | -2.19 | 0.423 | -0.339 | 0.049 |
|  | Apr vs Feb | -0.140 | 0.06 | -2.35 | 0.285 | -0.316 | 0.035 |
|  | May vs Feb | 0.064 | 0.06 | 1.01 | 1.000 | -0.122 | 0.249 |
|  | Apr vs Mar | 0.005 | 0.04 | 0.12 | 1.000 | -0.106 | 0.116 |
|  | May vs Mar | 0.208 | 0.04 | 5.13 | 0.000 | 0.089 | 0.328 |
|  | May vs Apr | 0.204 | 0.03 | 6.28 | 0.000 | 0.109 | 0.299 |

**
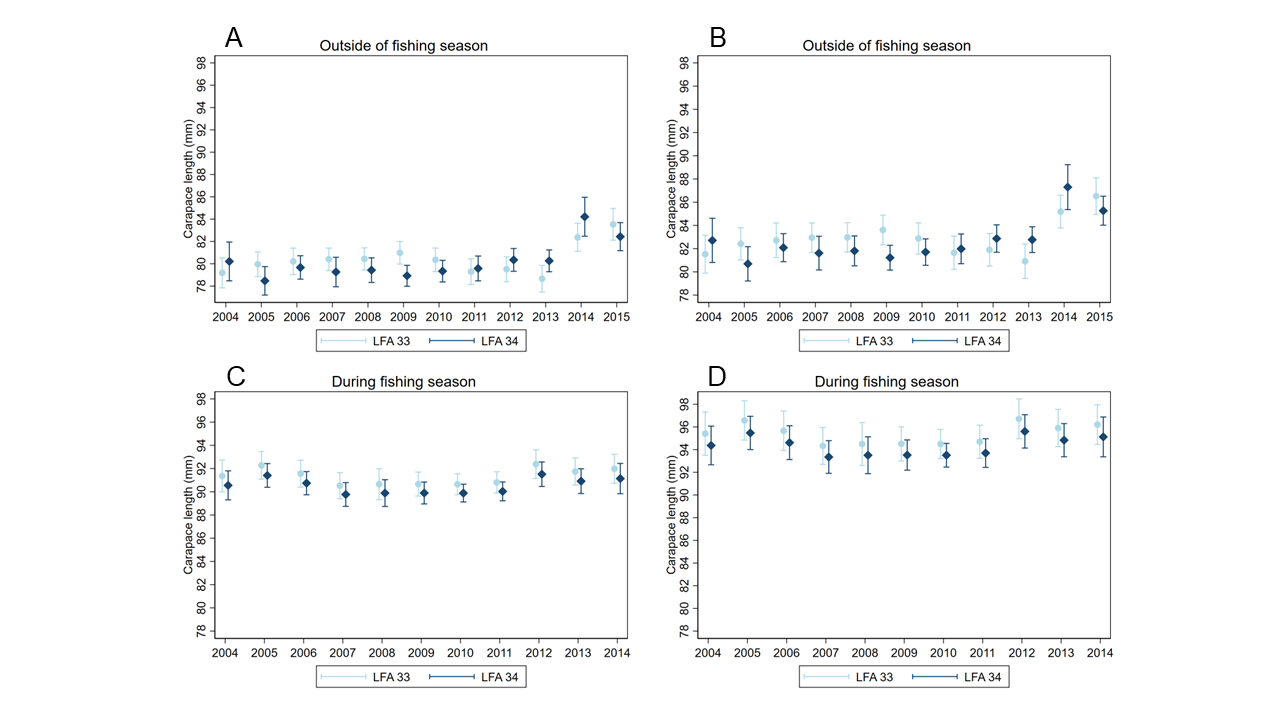
S1** **Figure. Estimated lobster sizes (with a 95% confidence interval) over the sampling period from 2004 to 2015 dependent on the LFAs outside the fishing season (OFS, Jun-Nov) and during the fishing season (DFS, Dec-May) from truncated regression models.** Model predictors not displayed on the graphs were fixed to: (A) month = June (OFS), sex = female, moult stage = intermoult, water depth = 10 m. (B) month = June (OFS), sex = male, moult stage = intermoult, water depth = 50 m. (C) month = May (DFS), sex = female, moult stage = intermoult, water depth = 10 m. (D) month = May (DFS), sex = male, moult stage = intermoult, water depth = 50 m. Note that there was no data available during the fishing season in 2015.

**
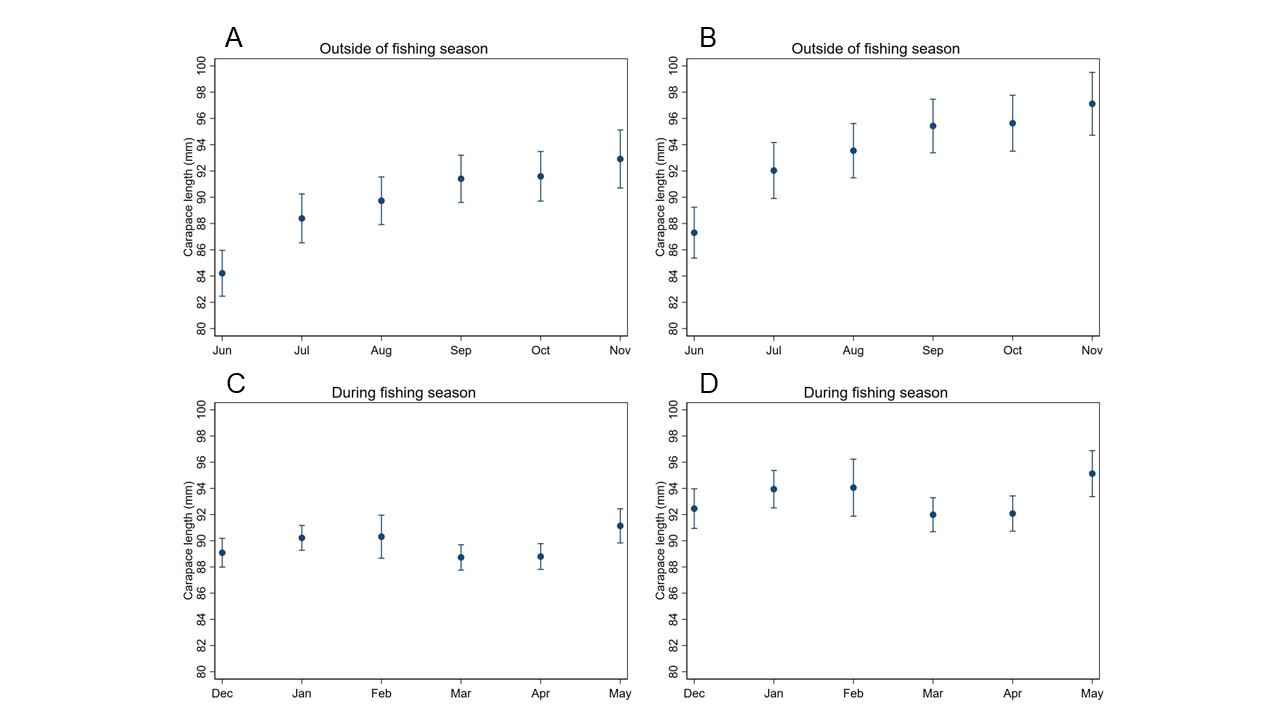
****S2 Fig. Estimated lobster sizes (with a 95% confidence interval) over the sampling year outside the fishing season (OFS, Jun-Nov) and during the fishing season (DFS, Dec-May).** Model predictors not displayed on the graphs were fixed to: (A) year = 2014, LFA = 34, sex = female, moult stage = intermoult, water depth = 10 m (B) year = 2014, LFA = 34, sex = male, moult stage = intermoult, water depth = 50 m, (C) year = 2014, LFA = 34, sex = female, moult stage = intermoult, water depth = 10 m, (D) year = 2014, LFA = 34, sex = male, moult stage = intermoult, water depth = 50 m.
